# Engineering biopolymer-based nanocarriers for cancer drug delivery: a system-level comparison of chitosan, hemicellulose, and lignin platforms with focus on tumor targeting, biodegradation, and immune modulation

**DOI:** 10.3389/fbioe.2026.1794222

**Published:** 2026-06-03

**Authors:** Li Wang, Yewei Liu, Bowen Wei

**Affiliations:** 1 Science and Technology College of Nanchang University, Jiujiang, China; 2 Jiangxi Agricultural University, Nanchang, China; 3 Independent Researcher, Jilin, China

**Keywords:** 5-fluorouracil, chitosan, drug delivery, hemicellulose, lignin

## Abstract

The emergence of biopolymer-based nanocarriers offers a transformative approach to cancer therapy, particularly in enhancing the efficacy and precision of chemotherapeutic agents such as 5-fluorouracil (5-FU), a widely used chemotherapeutic agent with limited tumor selectivity and rapid metabolic degradation. Among the vast array of natural polymers, chitosan, hemicellulose, and lignin, abundant, biodegradable, and derived from food and agricultural sources, have gained considerable attention as smart delivery vehicles. This review focuses on 5-FU as a clinically important but pharmacokinetically limited drug, and explores how polymer-based nanocarriers can overcome these challenges. We critically examine their biocompatibility, controlled release profiles, cellular uptake, and tumor-targeting mechanisms, along with their ability to overcome multidrug resistance and reduce systemic toxicity. Furthermore, we highlight recent advances in green synthesis techniques, surface engineering for active targeting, and synergistic applications with other bioactives or stimuli-responsive elements. By integrating insights from food science, nanotechnology, and oncology, this review underscores the promising role of chitosan, hemicellulose, and lignin nanoparticles in reshaping the landscape of cancer nanotherapeutics and paves the way for their translational potential in clinical settings.

## 1 Introduction

Cancer remains one of the leading causes of morbidity and mortality worldwide, necessitating the development of innovative therapeutic strategies to enhance treatment efficacy and minimize adverse effects. One such promising approach involves the use of nanocarriers for the targeted delivery of chemotherapeutic agents, such as 5-fluorouracil (5-FU) (Miura et al., 2010). 5-FU is a widely used antimetabolite that disrupts cancer cell proliferation by inhibiting thymidylate synthase, an essential enzyme in DNA synthesis (Longley et al., 2003). 5-FU is mostly used for treating solid tumors including breast, colorectal, and gastric cancers. Similar to every other chemotherapeutic drug, 5-FU has some unwanted effects on normal cells which causes several morbidities for patients who are treated with this drug. Despite its broad clinical use, 5-FU’s therapeutic potential is constrained by systemic toxicity, rapid degradation, and low tumor specificity, underscoring the need for advanced delivery approaches (Miura et al., 2010; Longley et al., 2003). Nanotechnology has entered the field of medicine for proving more targeted delivery systems which allow us to use chemotherapeutic drugs without worrying about their side effects. Recent advancements in nanotechnology have enabled the design of sophisticated nanoformulations that can further enhance the therapeutic index of 5-FU (Farokhzad and Langer, 2009; Masoud et al., 2025; Rata et al., 2021; Rață et al., 2019). These formulations can be engineered to respond to specific stimuli within the tumor microenvironment, such as pH or enzymatic activity, ensuring that the drug is released in a controlled manner where it is most needed. Additionally, surface modifications of chitosan nanoparticles can be employed to improve their circulation time and targeting capabilities, thereby increasing drug accumulation in tumor tissues (Farokhzad and Langer, 2009).

Chitosan, hemicellulose, and lignin are natural biopolymers with unique structural and functional properties that make them attractive for drug delivery applications. The selection of chitosan, hemicellulose, and lignin for this review is based on their distinct structural characteristics, physicochemical properties, and increasing relevance as sustainable biomaterials in drug delivery systems. These biopolymers represent three different classes of natural materials: chitosan as a cationic polysaccharide with well-established biomedical applications, hemicellulose as a heterogeneous plant-derived polysaccharide with tunable functionality, and lignin as a complex aromatic polymer with unique rigidity and antioxidant properties. This diversity enables a meaningful comparison of different mechanisms of drug encapsulation, release behavior, and biological interactions. Furthermore, all three have attracted growing attention in the development of nanocarriers for 5-FU, making them suitable candidates for a comparative analysis aimed at evaluating their relative advantages and limitations in anticancer drug delivery.

Chitosan, a biopolymer derived from chitin, has garnered significant attention in drug delivery systems due to its biocompatibility, biodegradability, and ability to form nanoparticles (Divya and Jisha, 2018). Its main limitations are poor solubility at neutral pH and batch-to-batch variability in molecular weight and deacetylation degree. Chitosan-based nanocarriers provide a versatile platform for encapsulating 5-FU, enhancing its solubility and stability while allowing for controlled release profiles. Moreover, cellulose and lignin with many similar effects and characteristics to chitosan can also be used for the same purpose (Dongsar et al., 2023). Hemicellulose, a heterogeneous polysaccharide obtained from plant cell walls and related to cellulose, is biodegradable, renewable, and chemically versatile, enabling the formation of hybrid nanostructures. However, its mechanical stability is generally lower than that of crystalline cellulose, and it may require chemical modification to achieve desired drug release properties. Lignin, an aromatic biopolymer abundant in lignocellulosic biomass, provides high antioxidant activity, UV stability, and multiple functional groups for chemical conjugation, making it suitable for stimuli-responsive systems. Its limitations include structural heterogeneity and low aqueous solubility. The rationale for focusing on these three polymers lies in their complementary properties, chitosan’s bioadhesion and permeability enhancement, hemicellulose’s versatility and processability, and lignin’s chemical functionality and intrinsic bioactivity, offering diverse and sustainable platforms for improving the delivery of 5-FU. The integration of nanocarriers based on chitosan, cellulose, and lignin for the delivery of 5-FU presents a compelling strategy to overcome the limitations associated with conventional chemotherapy. As research continues to explore the multifaceted benefits of this delivery system, it holds the potential to revolutionize cancer therapy and pave the way for more effective and safer treatment modalities.

Altogether, the clinical application of 5-FU is limited by rapid metabolism, poor tumor selectivity, and systemic toxicity. To address these limitations, this review focuses on the use of natural polymer-based nanocarriers, specifically those made from chitosan, hemicellulose, and lignin, as delivery platforms for 5-FU. These biopolymers offer advantages such as biocompatibility, biodegradability, and modifiable chemical structures that enable targeted, controlled drug release. By centering 5-FU as a model compound, this review aims to critically assess the therapeutic value of nanoformulations built on sustainable biopolymers in cancer therapy.

The literature for this review was retrieved from major scientific databases, including PubMed, Scopus, and Web of Science, using combinations of keywords such as “chitosan,” “hemicellulose,” “lignin,” “nanocarriers,” and “5-fluorouracil.” An overview of recent publication trends indicates a clear and sustained increase in research activity on biopolymer-based nanocarriers over the past 5 years. Among these, chitosan-based systems have been the most extensively studied, with a consistently higher volume of publications. In contrast, hemicellulose- and lignin-based nanocarriers have attracted growing attention more recently, although the overall body of literature remains comparatively limited. These trends reflect the differing levels of maturity and development across the three platforms.

## 2 5-Fluorouracil: from structure to mechanisms of action

### 2.1 The structure of 5-FU

Understanding the classification of chemotherapeutic drugs is essential to contextualize the role of 5-FU in cancer treatment. As an antimetabolite, 5-FU disrupts DNA synthesis by mimicking natural nucleotides, distinguishing it mechanistically from other drug classes such as alkylating agents or antimicrotubule agents. Including this classification helps illustrate the diverse mechanisms of action employed in chemotherapy and highlights the specific challenges associated with 5-FU, such as systemic toxicity, short half-life, and drug resistance. By establishing this foundation, the discussion underscores the rationale for developing advanced drug delivery systems, particularly biopolymer-based nanocarriers, that can overcome these limitations and enhance the clinical efficacy of 5-FU. As mentioned before, Fluoropyrimidines such as 5-FU were created after it was noted that rat liver tumors utilize the pyrimidine uracil more quickly than healthy tissues. This suggested that targeting uracil metabolism could be a viable approach for antimetabolite chemotherapy (Heidelberger et al., 1957). The development of 5-FU marked a pivotal advancement in the history of chemotherapy. First synthesized in 1957 by Charles Heidelberger and his team, 5-FU was designed based on the observation that cancerous liver tissues in rats exhibited a higher rate of uracil uptake than normal tissues (Heidelberger et al., 1957). By introducing a fluorine atom at the 5-position of the uracil ring, Heidelberger created a compound that could exploit this metabolic difference, leading to selective cytotoxicity in cancer cells. This innovation established 5-FU as one of the earliest examples of rational drug design and targeted cancer therapy. Since then, 5-FU has become a cornerstone in the treatment of a wide range of solid tumors, especially colorectal and breast cancers, and continues to be a subject of ongoing research for improving its delivery and reducing toxicity (Heidelberger et al., 1957).

**FIGURE 1:**
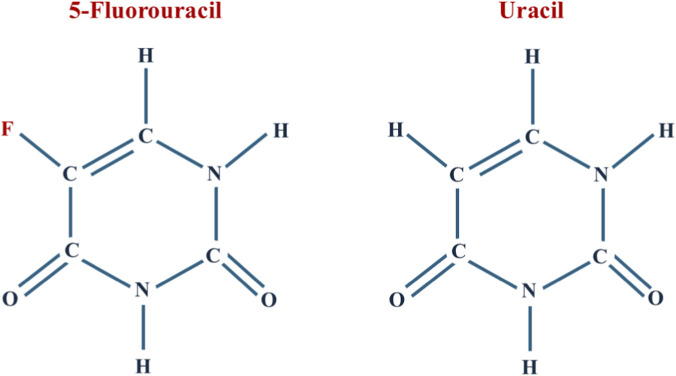
A schematic presentation of 5-Fluorouracil and uracil showing their similarities and differences.

5-FU is detected to be the analogue of uracil due to containing a ring similar to this nucleotide. The only difference between these two molecules is the replacement of a fluorine group on the carbon-5 position of the pyrimidine ring instead of a hydrogen in 5-FU (structures of both of these agents are shown in Figure 1). This synthetic pyrimidine analog has the chemical formula of C_4_H_3_F_3_N_2_O_2_. Its structure consists of a pyrimidine ring, which is a six-membered aromatic ring containing two nitrogen atoms at positions 1 and 3. The key features of 5-FU’s structure include a fluorine substitution, carbonyl and hydroxyl groups, and a pyrimidine backbone (Longley et al., 2003; Álvarez et al., 2012). A fluorine atom is substituted at the 5-position of the uracil ring which forms the fluorine Substitution and distinguishes it from uracil. This substitution is crucial for 5-FU anti-tumor activity. At the 2-position, there is a carbonyl (C=O) group, and at the 4-position, there is a hydroxyl (–OH) group. These functional groups contribute to the compound’s reactivity and biological activity. The core pyrimidine structure allows 5-FU to mimic uracil, enabling its incorporation into RNA and its interaction with enzymes involved in nucleic acid metabolism (Longley et al., 2003; Álvarez et al., 2012). The structural modifications of 5-FU compared to uracil play a critical role in its mechanism of action as a chemotherapeutic agent, particularly in inhibiting thymidylate synthase and disrupting DNA and RNA synthesis in cancer cells. Given the similarities in the structure of these two molecules, 5-FU quickly enters the cell through the same facilitated transport method as uracil. After entering cells, 5-FU needs to undergo some enzymatic alterations to transform into the nucleotide (involving ribosylation and phosphorylation) to exhibit its cytotoxic effects. There are two main mechanisms known for the conversion of 5-FU to nucleotides: anabolic mechanisms and catabolic mechanisms (Álvarez et al., 2012).

### 2.2 Catabolic mechanisms

An enzyme named dihydropyrimidine dehydrogenase (DPD) is considered as the basic step of the 5-FU catabolism. This enzyme is responsible for producing dihydrofluorouracil (DHFU) from 5-FU through reducing the double bond in the pyrimidine ring.in several organs which express this enzyme including liver (most importantly), mononuclear cells of blood, intestine, and lungs (Alzahrani et al., 2023). While DPD is the most researched enzyme in the catabolic pathway, there is no information available on the human version of this enzyme. The product produced by DPD is actually FUH2 which is unstable in water and may degrade on its own and therefore, another enzyme called dihydropyrimidinase converts FUH2 into 5-fluoroureidopropionic acid (FUPA). FUPA seems to be a temporary byproduct within the cell and is believed to be transformed into FBAL by the enzyme β-ureidopropionase (Miller, 1971).

### 2.3 Anabolic pathways

Similar to catabolism, the presence of fluorine does not hinder the anabolism of 5-FU through the pyrimidine pathway. There are three pathways through which 5-FU can be converted into cytotoxic nucleotides. The first process uses uridine phosphorylase and uridine kinase to convert it into the ribonucleotide 5-fluorouridine-5′-monophosphate (FUMP). The second process involves the direct transformation of 5-FU into FUMP through the action of orotidine-5′-monophosphate phosphoribosyltransferase. The third pathway, although less significant in terms of quantity, employs thymidine phosphorylase and thymidine kinase to convert 5-FU into the deoxyribosylnucleotide FdUMP (Miller, 1971; Ikenaka et al., 1979).

### 2.4 Mechanisms of action

While the exact mechanisms by which fluorouracil works are not completely understood, its primary action involves disrupting DNA synthesis and the translation of mRNA. In this section, we provide a summary of 5-FU mechanisms of actions.

#### 2.4.1 Inhibition of thymidylate synthase

The primary mechanism of action of 5-FU involves the inhibition of thymidylate synthase (TS), a critical enzyme in the *de novo* synthesis pathway of pyrimidine nucleotides. TS catalyzes the conversion of deoxyuridine monophosphate (dUMP) to deoxythymidine monophosphate (dTMP) using 5,10-methylenetetrahydrofolate as a cofactor. This reaction is essential for DNA synthesis, as dTMP is a precursor for thymidine triphosphate (dTTP), which is required for DNA replication and repair (Longley et al., 2003; Tanaka et al., 2000). 5-FU is metabolically converted into several active metabolites, most notably 5-fluoro-deoxyuridine monophosphate (FdUMP). FdUMP binds covalently to the active site of TS in the presence of reduced folate cofactor, forming a stable complex (Miura et al., 2010). This interaction effectively inhibits TS activity, leading to a depletion of dTTP levels. As a result, DNA synthesis is impaired, and cells are unable to proliferate normally. The inhibition of TS is particularly detrimental to rapidly dividing cancer cells, making them more susceptible to the cytotoxic effects of 5-FU (Longley et al., 2003; Tanaka et al., 2000).

#### 2.4.2 Incorporation into RNA

In addition to inhibiting TS, 5-FU can also be incorporated into RNA, where it interferes with RNA metabolism and function. Once inside the cell, 5-FU is phosphorylated to form various ribonucleotides. These metabolites can then be incorporated into RNA molecules during transcription, leading to the production of faulty RNA that cannot function properly (Miura et al., 2010). The incorporation of 5-FU into RNA affects several processes, including protein synthesis and splicing. By disrupting the normal function of RNA, 5-FU can further inhibit cell growth and proliferation. This mechanism is particularly relevant in tumors that are highly dependent on efficient RNA processing and translation for their growth (Longley et al., 2003; Tanaka et al., 2000).

#### 2.4.3 Induction of cell cycle arrest and apoptosis

The combined effects of TS inhibition and RNA interference lead to significant cellular consequences, including cell cycle arrest and apoptosis. The depletion of dTTP levels results in an imbalance in nucleotide pools, which triggers cell cycle checkpoints (Miura et al., 2010). Specifically, cells may experience G1 or S phase arrest due to the inability to synthesize DNA adequately. Prolonged cell cycle arrest can lead to cellular stress responses and ultimately result in programmed cell death (apoptosis) (Longley et al., 2003; Tanaka et al., 2000). Additionally, 5-FU has been shown to activate various apoptotic pathways. The accumulation of damaged DNA and misfolded proteins can activate p53, a tumor suppressor protein that plays a crucial role in regulating the cell cycle and apoptosis. The activation of p53 leads to the transcription of pro-apoptotic genes and the downregulation of anti-apoptotic factors, further promoting cell death (Longley et al., 2003; Tanaka et al., 2000).

#### 2.4.4 Synergistic effects with other agents

The efficacy of 5-FU can be enhanced when used in combination with other chemotherapeutic agents or supportive therapies. For instance, leucovorin (calcium folinate) is often administered alongside 5-FU to enhance its binding to TS. Leucovorin stabilizes the FdUMP-TS complex, resulting in prolonged inhibition of the enzyme and increased cytotoxicity against cancer cells. Moreover, combining 5-FU with other agents that target different pathways can provide synergistic effects. For example, when combined with oxaliplatin or irinotecan, 5-FU can enhance overall treatment efficacy in colorectal cancer by attacking multiple aspects of tumor biology simultaneously (Longley et al., 2003; Tanaka et al., 2000).

### 2.5 Pharmacology and pharmacokinetics of 5-FU

5-FU is primarily used in the management of various solid tumors, including colorectal, breast, stomach, and head and neck cancers. The drug exerts its therapeutic effects by interfering with the synthesis of nucleic acids, specifically by inhibiting the enzyme thymidylate synthase. This inhibition disrupts DNA synthesis and repair, leading to cell cycle arrest and apoptosis in rapidly dividing cancer cells. This drug can be administered as a single agent or in combination with other chemotherapeutic agents, such as leucovorin, which enhances its efficacy. The drug can be given via intravenous injection or infusion, as well as through topical application for certain skin cancers (Milano and Chamorey, 2002). The pharmacokinetics of 5-FU involves its absorption, distribution, metabolism, and excretion.

#### 2.5.1 Absorption

When administered intravenously, 5-FU is rapidly absorbed into the bloodstream. Oral formulations are less common due to variable absorption rates and first-pass metabolism.

#### 2.5.2 Distribution

5-FU is widely distributed throughout the body, including the liver, lungs, kidneys, and tumor tissues. The volume of distribution (Vd) is approximately 0.3–0.6 L/kg. The drug is known to cross the blood-brain barrier to some extent (Milano and Chamorey, 2002).

#### 2.5.3 Metabolism

5-FU undergoes extensive hepatic metabolism. It is primarily converted into several metabolites, including: Dehydro-5-FU, Fluoro-β-alanine, and Fluorouridine. Dehydro-5-the first metabolite is an active form that contributes to the drug’s anticancer effects while the second one is a non-cytotoxic metabolite, and the third one is another metabolite that has limited activity. As mentioned before, the metabolic pathways involve enzymes such as DPD, which plays a crucial role in the degradation of 5-FU. Genetic polymorphisms in DPD can lead to variations in drug clearance and toxicity among patients (Milano and Chamorey, 2002).

#### 2.5.4 Excretion

The elimination half-life of 5-FU ranges from 8 to 20 min due to rapid clearance from the bloodstream. The drug and its metabolites are primarily excreted via the urine. Renal function can significantly affect the clearance of 5-FU; thus, dose adjustments may be necessary in patients with impaired renal function (Milano and Chamorey, 2002).

## 3 Drug delivery systems

**FIGURE 2:**
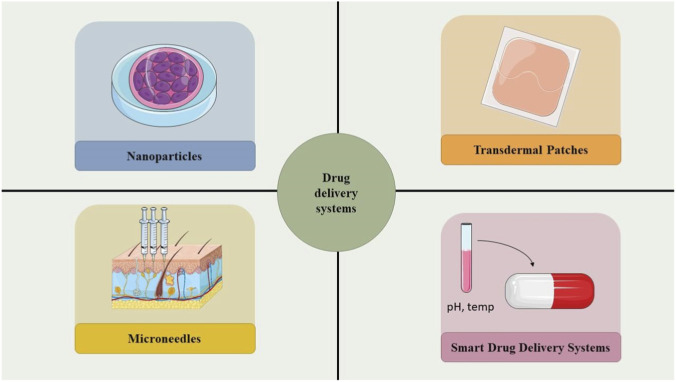
A summary of drug delivery systems.

Drug delivery systems (DDS) play a crucial role in modern medicine by determining the way a drug is administered, its pharmacokinetics, and ultimately its therapeutic efficacy. The development of effective drug delivery systems is essential for optimizing the therapeutic effects of drugs while minimizing side effects. Drug delivery refers to the method or process of administering a pharmaceutical compound to achieve a therapeutic effect in humans or animals (Tiwari et al., 2012) (Figure 2).

The primary goals of drug delivery systems include:

Targeted Delivery: Delivering the drug to a specific site in the body to enhance efficacy and reduce side effects.

Controlled Release: Regulating the release rate of the drug over time to maintain optimal therapeutic levels.

Improved Bioavailability: Enhancing the absorption and distribution of drugs to ensure sufficient concentrations at the site of action (Tiwari et al., 2012).

As science progresses, new and varied types of DDS are also provided. Conventional drug delivery systems include traditional methods such as oral, intravenous, intramuscular, and subcutaneous routes. Each route has distinct characteristics; for instance, oral delivery is the most common route which can be limited by factors like first-pass metabolism and variable absorption rates. Intravenous delivery Provides immediate drug action and precise control over dosage but requires skilled personnel for administration. And the last but not least, intramuscular and Subcutaneous Delivery: Useful for depot formulations that provide sustained release but may cause discomfort or pain at the injection site (Jain, 2008; Kshirsagar, 2000).

Besides these systems, novel drug delivery systems are also provided for achieving a better efficacy of drugs in specific diseases. Advanced drug delivery systems aim to improve upon conventional methods by incorporating new technologies and materials. Nanotechnology is one of the pioneers in this field which have helped providing a wide range of systems. Nanoparticles are engineered materials ranging from 1 to 100 nm in size. They can encapsulate drugs, improving solubility, stability, and bioavailability. There are three main types of nanoparticles; liposomes, polymeric nanoparticles, and metallic nanoparticles (Deng et al., 2020; Wilczewska et al., 2012). Liposomes are spherical vesicles that can encapsulate hydrophilic or hydrophobic drugs, enhancing their delivery and reducing toxicity. Polymeric nanoparticles are Biodegradable polymers that can provide controlled release and targeted delivery, and metallic nanoparticles are Gold or silver nanoparticles that can be used for imaging and therapy (Patra et al., 2018).

Microneedles. These systems are minimally invasive devices that penetrate the skin’s outer layer to deliver drugs into the dermis. They can be used for vaccines, insulin, and other biologics, offering pain-free administration and improved patient compliance.

Transdermal Drug Delivery Systems. These are another system which allow for the continuous release of drugs through the skin over an extended period. They are particularly useful for delivering hormones, analgesics, and nicotine.

Smart Drug Delivery Systems. Smart delivery systems are capable of responding to physiological changes (e.g., pH, temperature) could provide more precise control over drug release profiles (Deng et al., 2020; Wilczewska et al., 2012).

## 4 Fluorouracil and chitosan-based delivery systems

**FIGURE 3:**
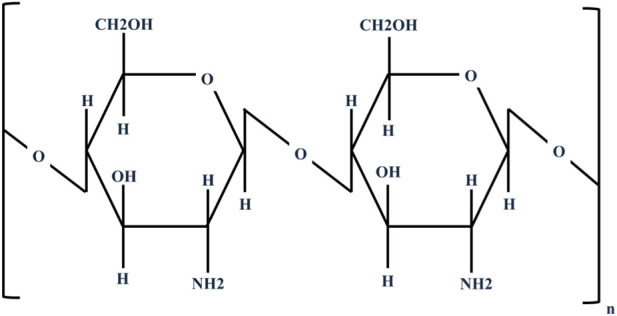
Chitosan structure. Chitosan is composed of repeating units of N-acetyl-D-glucosamine and D-glucosamine. The structural formula for these monomers is: N-acetyl-D-glucosamine (GlcNAc): C8H15NO6, D-glucosamine (GlcN): C6H13NO5, The presence of amino (-NH2) and hydroxyl (-OH) functional groups in chitosan contributes to its chemical reactivity and solubility.

There is a great body of evidence investigating the efficacy of using chitosan-based nanocarriers for delivering 5-FU to various cancer cells including brain tumors, skin cancer, osteosarcoma, etc. (Dellali et al., 2022). In this section we review all the investigations which tried to provide a more efficient way for delivering 5-FU by the means of chitosan (Table 1; Figure 3).

**TABLE 1:** Studies investigating the effectiveness of chitosan-based nano-carriers for delivering 5-FU to diverse cancer cells.

Type of cancer	Model of study	Population/Cell line	Carrier	Result(s)	References
Melanoma	*In vitro*	B16F10 cancer cells	Chitosan-based tripolyphosphate-crosslinked pH-sensitive niosomal nanogels	Increasing anticancer effectiveness and reducing toxicity to normal cells, a slow release of 5-FU	Ahmed et al. (2023)
Osteosarcoma	*In vitro*	HOS cells, MG-63 cells	Chitosan-based microspheres	Increasing the absorption of 5-FU and thereby, enhancing its effects	Zhao et al. (2022)
Glioblastoma	*In vitro*	U87 cells	0.2% chitosan-Selenium Nanoparticles	Increasing the sensitivity of 5-FU against glioma cells and decreasing cell invasion and migration	Dana et al. (2022)
Colorectal cancer	*In vitro*	HT-29 cells	chitosan-1-acetic acid-5-fluorouracil conjugates	Increasing half-life, stability, and effectiveness	Mohammed et al. (2017)
	*In vitro*	HT-29 cells	enteric-coated PEG-cross-linked chitosan microspheres	Improving safety profile of 5-FU	Ganguly et al. (2016)
	*In vivo*	Rats	enteric-coated PEG-cross-linked chitosan microspheres	Increasing drug concentration in liver and drug efficacy	Ganguly et al. (2016)
	*In vitro*	HCT-116 cancer cells	chitosan nanoparticles conjugated with methotrexate	Enhancing cytotoxic effects after 24 h with IC50 values lower than 27.44 μg/mL	Dos Santos et al. (2024)
	*In vivo*	CRPC mouse model	thermosensitive Chitosan hydrogels	Enhancing drug anti-tumor effects and decreasing metastasis to liver and lung	Yun et al. (2017)
	*In vitro*	HCT116 colorectal cancer	Chitosan-Cellulose Fiber Bionanocomposites	eliminating 56.42% ± 0.41% of cancer cells and only 8.16% ± 2.11% of normal cells and enhancing drug encapsulation efficacy	Yusefi et al. (2021a)
Breast cancer	*In vitro*	MCF-7 human breast cancer cells	iron-based chitosan-coated MIL-100 composite	More cytotoxic effects on cancer cells and less on normal cells	Resen et al. (2022)
	*In vitro*	PANC-1 and MDA-MD 231	aptamer-conjugated chitosan-bimetallic nanoparticles	​	Saravanakumar et al. (2024)
	*In vitro*	MCF-7 cell lines	Chitosan, aptamer, and carbon quantum dot (CS/Apt/COQ) hydrogels	Increasing entrapment efficacy and prolonged drug release	Mazandarani et al. (2023)
	*In vitro*	MCF-7 cell lines	Chitosan hydrogels	Increasing the suppression of cancer cell viability	Abdellatif et al. (2022)
	*In vivo*	Female rats	Chitosan hydrogels	Reducing tumor volume and tumor markers and 5-FU side effects	Abdellatif et al. (2022)

### 4.1 Breast cancer

Breast cancer is one of the most common cancers worldwide, affecting millions of women and, to a lesser extent, men. According to the World Health Organization (WHO), it accounts for approximately 25% of all cancer cases in women, making it the most prevalent cancer globally (Barzaman et al., 2020). The incidence of breast cancer varies significantly by region, with higher rates observed in developed countries compared to developing nations. Factors contributing to this variation include differences in lifestyle, reproductive patterns, and access to healthcare. Mortality rates from breast cancer have seen a decline in many high-income countries, largely due to advancements in early detection through screening and improvements in treatment options. However, breast cancer remains a leading cause of cancer-related deaths among women, with an estimated 685,000 deaths globally in 2020. In low- and middle-income countries, late-stage diagnosis and limited access to treatment contribute to higher mortality rates. Given this, it’s important to develop more therapeutic options for decreasing the burden of this cancer (Barzaman et al., 2020; Harbeck and Gnant, 2017).

A study on breast cancer cells shows that using a nano-carrier produced based on MIL-100 nanocomposite can be effective for enhancing the efficacy of 5-FU. They encapsulated 5-FU inside the MIL-100 and coated this combination with chitosan and chelated it with iron (III) and then added quercetin at the final step. Their multifunctional MIL-100 nanocarrier, which could release 5-FU in a pH-sensitive manner, was able to induce apoptosis in human breast cancer cells. They also identified that chitosan-coated 5-Fluorouracil@MIL-100 with quercetin showed greater effectiveness in MCF-7 cells compared to other nanocarriers (P < 0.05) while they had a lesser cytotoxic effect on normal cells (Resen et al., 2022). Another research group also sought to develop multifunctional aptamer-conjugated chitosan-bimetallic (Au/Pd) nanoparticles loaded with photothermally responsive 5-FU (APT-CS-5-FU-Au/Pd NPs) to enhance cytotoxic effects on two cancer cell lines, PANC-1 and MDA-MB 231 (Saravanakumar et al., 2024). Their nano-carriers had the size of 34.43 ± 1.59 nm and increased the release of 5-FU (Saravanakumar et al., 2024). They observed that at pH 5.4 after 74 h, a greater release of 5-FU (69.8% ± 2.78%) was noted. NIR-APT-CS-5-FU-Au/Pd nanoparticles did not exhibit toxicity towards RBC and the egg chick chorioallantoic membrane (CAM), but they enhanced cytotoxicity in MDA-MB 231 and PANC-1 cells by inducing oxidative stress-related cell death (Saravanakumar et al., 2024). Another study also tried to provide a sustained release for 5-FU on breast cancer cells through the combination of Chitosan, aptamer, and carbon quantum dot (CS/Apt/COQ) hydrogels (Mazandarani et al., 2023). They provided a pH-sensitive delivery system with an average size of 250.6 nm and a polydispersity index (PDI) of 0.057 and an average surface charge of +37.8 mV (Mazandarani et al., 2023). Loading 5-FU into these nanoparticles showed that at pH 5.4, there was a highly effective and sustained release of the drug, with nearly all of the 5-FU and GA being released within 48 h. The entrapment efficiencies for 5-FU and GA were 84.7% ± 5.2% and 80.2% ± 2.3%, respectively. The combination of 5-FU-GA-CS-CQD-Apt resulted in the most significant cell death, leaving only 57.9% of the MCF-7 cells alive after treatment. Additionally, 5-FU and GA in CS-CQD-Apt increased apoptotic induction as measured by flow cytometry. The 5-FU-GA-CS-CQD-Apt combination also raised the levels of Caspase 9 while decreasing the levels of Bcl2 (Mazandarani et al., 2023). Similarly, Abdellatif and colleagues also tried chitosan hydrogels for delivering 5-FU to MCF-7 cells (Abdellatif et al., 2022). Their altered hydrogel possesses outstanding physicochemical characteristics, exhibiting a stable *in vitro* release pattern that aligns with zero-order kinetics over the course of a month. Furthermore, the hydrogel demonstrated significantly better suppression of cell viability compared to the untreated control group (Abdellatif et al., 2022). They performed an *in vivo* investigation by injecting these hydrogels into breast cancer cells of female rats (intratumor injection) and found that these hydrogels have effective antitumor activity with minimal side effects. Compared to the untreated control group, there was a significant reduction in tumor volume and tumor marker levels in the blood when the hydrogels was used (Abdellatif et al., 2022).

### 4.2 Colorectal cancer

Colorectal cancer (CRC) is a significant global health concern and ranks as the third most common cancer and the second leading cause of cancer-related deaths worldwide. According to the World Health Organization (WHO), there were approximately 1.9 million new cases and 935,000 deaths from colorectal cancer in 2020 (WHO, 2023). Mortality rates from colorectal cancer are notably high, particularly in low- and middle-income countries where access to screening and treatment may be limited. The 5-year survival rate can exceed 90% when detected early; however, late-stage diagnosis significantly reduces survival rates (WHO, 2023). There are plenty of reasons which make CRC a cancer with poor prognosis; for instance, late detection and the presence of cancer cells resistant to multiple drugs are the main reasons however, cancer recurrence, metastasis, and the invasive nature of CRC cells are also responsible for its poor prognosis. There are some *in vitro* and *in vivo* studies which tried to target CRC cells by chitosan-based nanoparticles. One of these studies is conducted by Ganguly et al. (2016) which created Chitosan (CS) microspheres that are cross-linked with polyethylene glycol (PEG) using an emulsion-cross-linking process, followed by a solvent evaporation method. They used these microspheres *in vitro* on human HT-29 colon cancer cell-line and found out that the growth of tumor cells was suppressed to trigger apoptosis over a prolonged period after. The minimum inhibitory concentration (IC50) for standard plain 5-FU was 5.00 ± 0.004 μg/mL, while for 5-FU-loaded microspheres it was 165 ± 1.9 μg/mL, indicating that the microsphere formulation has a better safety profile (Ganguly et al., 2016). They also used this method *in vivo* on rats and assessed tissue distribution of their carrier. The tissue distribution revealed that the concentration of 5-FU in the colon exceeded the IC50 value necessary to halt the proliferation or induce death in colon cancer cells associated with colonic dysplasia. When animals were treated with the standard formulation of 5-FU, a notable decrease in tumor volume and number was seen, along with elevated levels of liver enzymes, compared to those treated with 5-FU loaded microspheres (Ganguly et al., 2016). The most recent study in this field in performed in 2024, by Dos Santos et al. (2024) which designed chitosan-based nanoparticles conjugated with methotrexate (MTX) for delivering 5-FU. These nanoparticles had a particle size between 376.4 and 407.8 nm, and demonstrated a high positive zeta potential, and achieved an encapsulation efficiency for 5-FU exceeding 15%. After an *in vitro* utilization, it was shown that the mucoadhesive ability of chitosan can be decreased, allowing for more targeted interactions with tumor cells. Both unmodified and MTX-modified nanoparticles loaded with 5-FU showed greater cytotoxic effects on HCT-116 cancer cells after 24 h than the free drugs, with IC50 values lower than 27.44 μg/mL (Dos Santos et al., 2024).

Another study transformed 5-FU into 5-fluorouracil-1-acetic acid (FUAC) and combined with chitosan to create chitosan-1-acetic acid-5-fluorouracil (CS-FUAC) conjugates, which were subsequently evaluated for use in colon cancer treatment (Mohammed et al., 2017). The conjugates that were prepared demonstrated greater stability under basic conditions. Notably, these conjugates had a longer half-life than the original drug. An *in vitro* cytotoxicity study indicated that these products are more effective than the free drug. Specifically, the *in vitro* study revealed that these prodrug conjugates are more effective against human colorectal cancer cell lines (HT-29) than the unmodified drug. Additionally, these conjugates were nearly twice as toxic to the colon cancer cells compared to the normal cells (Mohammed et al., 2017). Studies on chitosan hydrogels also show that administering via intraperitoneal injection can reduce tumor growth and metastasis, as well as extend survival time compared to other groups, thereby enhancing the effectiveness of chemotherapy. Ki-67 immunohistochemical analysis shows that tumors treated with the CS hydrogel drug exhibit decreased cell proliferation compared to the other groups (P < 0.001) (Yun et al., 2017). In addition, staining liver and lung tissue with hematoxylin-eosin shows that the CS hydrogel drug has a notable inhibitory effect on the spread of colorectal cancer to these organs (Yun et al., 2017). Interestingly, along with nano-carriers which are provided only with chitosan, there are some other carriers which are created by the combination of chitosan with another biocompatible polymer like cellulose and has also showed advantageous effects. For instance, Yousefi and colleagues used the ionic gelation method and synthesized three different chitosan-based carriers: chitosan-cellulose fibers bionanocomposites (CS-CF/5-FU BNCs), chitosan-cellulose fibers bionanocomposites (CS-CF BNCs), and chitosan nanoparticles (CS NPs). They utilized a 1:2 ratio of cellulose and chitosan to create a spherical nanocomposite with a size of less than 50 nm, as shown in the SEM images (Yusefi et al., 2021a). They observed that the drug release time was recorded at 8 h, resulting in percentages of 50.60% ± 1.88% in fluid with pH 7.4% and 28.04% ± 1.14% in fluid with pH 1.2. After 36 h, the total release of 5-FU was lower in the acidic environment (pH 1.2), measuring at 42.37% ± 0.43%, compared to the simulated colorectal fluid at pH 7.4, which was 76.82% ± 1.29%. This difference may be attributed to the fact that CS-CF/5-FU BNCs decreased in size in the acidic medium (Yusefi et al., 2021a). Their cytotoxicity assays also showed that a concentration of 15.62 μg/mL of chitosan nanoparticles was sufficient to eliminate 26.46% ± 6.52% of CRC cells while exhibiting no toxicity towards normal cells. Furthermore, the maximum concentration (500 μg/mL) of cellulose, chitosan nanoparticles, and CS-CF BNCs demonstrated good biocompatibility, with no harm to normal cells. Among the samples that did not include the loaded-5-FU, CS-CF BNCs showed a superior anticancer effect of 41.62% ± 3.15%, indicating an effective collaboration between CS and CF in the composites (Yusefi et al., 2021a). Specific concentrations of CS-CF/5-FU BNCs caused minimal harm to normal cells while showing significant anti-proliferative effects against cancer cells. In particular, the CS-CF/5-FU BNCs at a concentration of 250 μg/mL were able to kill 56.42% ± 0.41% of cancer cells, with only 8.16% ± 2.11% of normal cells being affected. This anticancer effect may be related to the unique properties of the synthesized double polysaccharides, which may enhance the encapsulation of 5-FU, thereby improving its mobility and binding capabilities (Yusefi et al., 2021a).

### 4.3 Other cancers

One of the most recent studies has tried to determine the efficacy of 5-FU-loaded niosomes on (Ahmed et al., 2023). A niosome is a lipid-based vesicle made from nonionic surfactants that typically has one hydrophobic tail. Ingredients like terpenoids, polysorbates, alkyl oxyethylenes, and spans are employed to create niosomes. In this study, niosomes were prepared through conventional thin film hydration technique which in summary, Span 80, Tween 80, and cholesterol were mixed in 5 mL of chloroform and methanol (in a 3:2 ratio) while stirring continuously (Ahmed et al., 2023). After the substances were fully dissolved, the mixture was placed into a 50 mL round-bottom flask containing glass pearl beads and rotated in a rotary evaporator at a temperature of 60 °C–65 °C under vacuum until a thin film developed on the flask’s walls. Afterwards, the dried thin film was transferred to a vacuum oven set at 50 °C for 2 h (Ahmed et al., 2023). Following this, the thin film was soaked in 10 mL of deionized water mixed with various concentrations of 5-Fu (20%, 40%, and 60% relative to cholesterol content) while being stirred gently for 1 h to create 5-FU-loaded niosomes. In the end, to coat the resulting niosomes with chitosan, 1 mg/mL of low molecular weight chitosan was mixed into 0.5% acetic acid while stirring continuously. Next, the prepared niosomes were gradually added to the chitosan solution, followed by the addition of 2 mL of a 0.5 mg/mL TPP solution to create nanochitosan-coated niosomal nanogels (Ahmed et al., 2023). The results of this shows that the niosomal nanocarriers containing 5-FU in this study produced formulations characterized by small nanoscale particle sizes, a semi-spherical shape, and relatively favorable % yield, % efficiency, and % loading. The Ch-coated formulations exhibited marginally smaller particle sizes and a positive zeta potential (Ahmed et al., 2023). *In vitro* drug release experiments demonstrated that 5-FU was released at a slower rate from the Ch-coated and TPP-crosslinked formulations compared to the uncoated niosomal nanoparticles. All the formulations tested exhibited hemocompatibility in hemolysis assays, showing a hemolysis percentage of less than 5% at their maximum concentrations (500 μM and 1 mM) (Ahmed et al., 2023). The formulations demonstrated greater anticancer effectiveness against B16F10 cancer cells and reduced toxicity to NIH3T3 normal cells compared to both the control and pure 5-FU within the tested concentration range of 10–100 µM. Furthermore, the examination of the cell migration inhibition effects of the developed formulations showed results that were consistent with the *in vitro* cell viability tests (Ahmed et al., 2023).

Another study tried chitosan microspheres which were provided with a 1.5 wt% chitosan solution (Mw = 100,000). Then for the production of drug-releasing chitosan microspheres, 50 mg each of 5-FU, PTX, and CDDP were dissolved in 50 mL of DMF. Next, 1.5 wt% chitosan and 2% acetic acid were added to this mixture. The solution underwent sonication for 30 min to ensure it was homogenous before the electrospraying process (Ahmed et al., 2023). The chitosan microspheres in this study had a consistent size, with an average measurement of 532 μm. Most of them fell within the range of 450–650 μm, representing 77.77% of the total. Zhao and colleagues suggested that the size of the resulting microspheres can be modified by altering the concentration of the solution being electrosprayed and the voltage applied to the droplets. Furthermore, larger average sizes allow for the encapsulation of more drugs or larger molecules within the carrier, a feat that is difficult to accomplish with other nanoparticles such as liposomes (Zhao et al., 2022). Analysis on these microspheres showed that 5-FU encapsulation efficiency of these microspheres was 72.4% ± 4.3%; furthermore, the release of 5-FU started off rapidly and reached completion in approximately 10 days (Zhao et al., 2022). This study showed that chitosan microspheres can increase the absorption of 5-FU and thereby, inhibit the growth and migration and enhance apoptosis of osteosarcoma cells *in vitro* (Zhao et al., 2022).

Dana and colleagues also tried to provide a chitosan-based nanoparticle and try its efficacy on glioblastoma cells (Dana et al., 2022). They synthesize Selenium NPs, a 1 mL solution of 0.1 M sodium selenite was created, followed by the addition of a 0.1 M L-ascorbic acid solution. They dissolve 10 mL of chitosan at a concentration of 0.10%–0.20% w/v in 85 mL of distilled water using magnetic stirring at 700 rpm for 30 min and then, gradually introduce 1 mL of 0.1 M sodium selenite solution into the mixture (Dana et al., 2022). Their results showed that these synthetic NPs had spherical shapes with sizes under 100 nm (Dana et al., 2022). The sizes of chitosan-based selenium NPs (Cs-SeNPs) containing chitosan concentrations of 0.1%, 0.15%, and 0.2% measured 88.66 ± 0.65 nm, 88.90 ± 0.57 nm, and 91.45 ± 2.57 nm, respectively. Therefore, there was no significant difference in the sizes for the various chitosan concentrations in Cs-SeNPs (Dana et al., 2022). 0.2% CS-SeNPs exhibited a significant difference in toxicity between normal cells and cancer cells. At the maximum concentration of 25 μg/mL, 0.2% CS-SeNPs resulted in the lowest cell viability compared to other percentages of CS-SeNPs. In contrast, normal fibroblasts were the least affected (Dana et al., 2022). The findings from RT-PCR suggest that a concentration of 0.2% Cs-SeNPs can increase the sensitivity of 5-FU against glioma cells by reducing the expression of MRP1 and plus, decreases cell migration and Invasion of these cells (Dana et al., 2022). Resen and colleagues also provided a nano-carrier based on chitosan in order to increase the efficacy of 5-FU on breast cancer cells (Resen et al., 2022).

**FIGURE 4:**
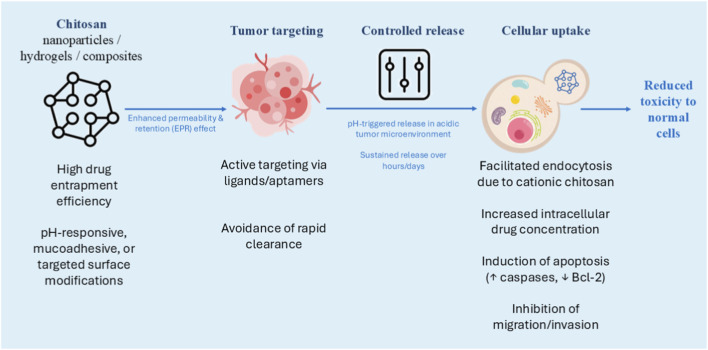
Mechanism of action and safety benefits of chitosan-based 5-fluorouracil (5-FU) delivery systems. Chitosan nanocarriers improve drug entrapment efficiency, enable tumor-targeted delivery via enhanced permeability and retention (EPR) effect or active targeting, provide pH-triggered and sustained release, increase intracellular drug concentration in tumor cells, induce apoptosis, and reduce systemic and off-target toxicities.

Across the reviewed studies, chitosan-based nanocarriers consistently enhanced the anticancer performance of 5-FU by improving drug stability, prolonging release, and increasing tumor selectivity. Reported mechanisms included pH-responsive or mucoadhesive release, improved cellular uptake, induction of apoptosis, and inhibition of cancer cell migration and invasion. *In vitro*, these carriers often achieved markedly higher cytotoxicity toward cancer cells while sparing normal cells, for example, chitosan–cellulose bionanocomposites eliminated 56.4% of colorectal cancer cells but affected only 8.2% of normal cells, and aptamer-conjugated chitosan nanoparticles induced strong oxidative stress in tumor cells without harming red blood cells or the chick chorioallantoic membrane. *In vivo* findings also indicated reduced systemic toxicity compared with free 5-FU, such as lower elevations of liver enzymes and fewer hematologic side effects, alongside effective tumor suppression and decreased metastasis. Collectively, the data support that chitosan-based carriers not only potentiate the therapeutic action of 5-FU but also improve its safety profile through selective delivery and reduced off-target effects (Figure 4).

Overall, the reported effects of chitosan-based nanocarriers vary among different cancer types, reflecting both biological differences between cell lines and the mechanisms governing nanoparticle uptake. The largest number of studies has been conducted in colorectal cancer models such as HT-29 and HCT-116, where chitosan-mediated delivery of 5-FU consistently demonstrates enhanced cytotoxicity and improved intracellular drug accumulation compared with the free drug. This relatively large body of evidence provides stronger statistical reliability for colorectal systems, likely due to the established clinical relevance of 5-FU in colorectal cancer therapy. In breast cancer models, particularly MCF-7 cells, chitosan nanoparticles also show improved antiproliferative activity; however, the magnitude of the effect is often influenced by nanoparticle size, surface modification, and drug-loading efficiency. Studies involving cervical cancer cells such as HeLa similarly report increased cellular uptake and apoptosis induction, although the number of investigations remains smaller than in colorectal models. Mechanistically, these differences are primarily associated with variations in membrane composition, receptor expression, and endocytic activity across cancer cell types, which influence how efficiently chitosan nanoparticles interact with and enter the cells. Consequently, while chitosan-based carriers consistently enhance the delivery of 5-FU across multiple cancer models, the strength of evidence and the magnitude of therapeutic response appear to be most robust in colorectal cancer systems, where both the biological sensitivity to 5-FU and the number of experimental studies provide stronger support for their effectiveness.

### 4.4 Challenges and opportunities in chitosan-based 5-FU delivery systems

Despite the growing body of research supporting the application of chitosan-based nanocarriers for 5-FU delivery, several critical challenges must be addressed to facilitate clinical translation. One of the primary limitations lies in the heterogeneity of synthesis protocols, which results in considerable variability in particle size, surface charge, drug loading efficiency, and release kinetics. This lack of standardization complicates the comparison of findings across different studies and hinders the development of universally applicable formulations. Moreover, while many studies have demonstrated promising cytotoxicity and sustained release profiles *in vitro*, there remains a significant gap in *in vivo* validation, particularly in clinically relevant animal models and human trials (Duceppe and Tabrizian, 2010; Iacob et al., 2021; Manna et al., 2023).

Another challenge is the incomplete understanding of the interaction between chitosan nanoparticles and the biological milieu. Factors such as the immune response, protein corona formation, and nanoparticle clearance via the mononuclear phagocyte system are rarely addressed in the literature, yet they play a critical role in determining biodistribution and therapeutic efficacy. Additionally, while chitosan is generally regarded as biocompatible, its degree of deacetylation, molecular weight, and the presence of residual solvents or cross-linkers can influence its safety and pharmacokinetic profile, raising concerns about reproducibility and regulatory approval (Duceppe and Tabrizian, 2010; Iacob et al., 2021; Manna et al., 2023). Despite these obstacles, there are significant opportunities for advancing chitosan-based 5-FU delivery systems. The polymer’s inherent mucoadhesive properties, pH-sensitivity, and ability to be chemically modified offer avenues for creating targeted, stimuli-responsive systems capable of site-specific drug release. Furthermore, chitosan can be easily conjugated with ligands or other polymers to enhance tumor selectivity and overcome multidrug resistance. With continued focus on optimizing formulation parameters, improving biological characterization, and conducting rigorous preclinical testing, chitosan-based nanocarriers hold strong promise for transitioning from laboratory research to viable clinical applications in cancer therapy (Duceppe and Tabrizian, 2010; Iacob et al., 2021; Manna et al., 2023).

## 5 5-Fluorouracil and hemicellulose-based delivery systems

**FIGURE 5:**
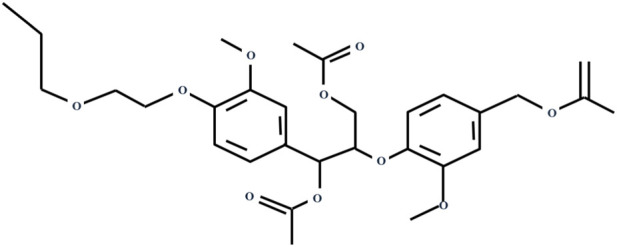
Lignin structure. Lignin structure is primarily formed from three main aromatic alcohols, known as monolignols: P-coumaryl alcohol (H-unit), Coniferyl alcohol (G-unit), Sinapyl alcohol (S-unit). These monolignols are linked together via a series of ether and carbon-carbon bonds, resulting in a three-dimensional network.

Hemicellulose, a major component of plant cell walls, has gained attention in the field of drug delivery due to its biocompatibility, biodegradability, and ability to form hydrogels. Its unique structural properties allow it to encapsulate various therapeutic agents, including proteins, peptides, and small molecules, enhancing their stability and controlled release (hemicellulose structure is shown in Figure 5).

In drug delivery applications, hemicellulose can be modified to improve its solubility and functionality, enabling targeted delivery to specific tissues or cells. For instance, hemicellulose-based nanoparticles can be engineered to release drugs in response to specific stimuli, such as pH changes or enzymatic activity, making them suitable for localized treatment of diseases like cancer. Moreover, hemicellulose can be combined with other biopolymers to create composite materials that exhibit synergistic effects, further improving drug encapsulation efficiency and release profiles. Its natural origin also aligns with the growing demand for sustainable and eco-friendly materials in pharmaceuticals. Regarding the delivery of 5-FU, there are some evidence indicating the effectiveness of hemicellulose-based nano-carriers. These studies are reviewed below and are summarized in Table 2.

**TABLE 2:** Studies investigating the effectiveness of cellulose-based nano-carriers for delivering 5-FU to diverse cancer cells.

Type of cancer	Model of study	Population/Cell line	Carrier	Result(s)	References
Breast cancer	*In vitro*	MCF-7	Nanocomposite based on halloysite nanotube (HNT) coated with carboxymethyl cellulose/polyethylene glycol hydrogel	Increasing drug entrapment and loading (46% and 87%, respectively)	Ghasemizadeh et al. (2023)
	*In vitro*	MCF-7	PVP/CMC/γ-alumina nanocomposite	controlled drug release behavior with above 96 h and above 90% cell viability	Shamsabadipour et al. (2023)
	*In vitro*	ZR 75-1 and MDA/MB 231	cellulose nanofibers combined with alginate/pectin	Enhancing cytotoxic effects of 5-FU	Balahura et al. (2021)
	*In vitro*	MDA-MB 231	nanofiber mats composed of polylactic acid, methylcellulose, polyethylene glycol and iron oxide nanoparticles	Increasing drug encapsulation efficacy and anti-cancer effects	Jaisankar et al. (2024)
	*In vitro*	MDA-MB 231	Fabricate polycaprolactone-based nanofiber mats involving MgO nanoparticle, methyl cellulose, and polyethylene glycol	Controlling drug release pattern	Jaisankar et al. (2022)
Colorectal cancer	*In vitro*	2D monolayer and 3D tumour spheroid models	Fe3O4 nanofillers covered the cellulose matrix	Increasing cytotoxicity and selectivity of 5-FU	Yusefi et al. (2021b)
	*In vitro*	HT-29 colon cancer cell	polyhydroxybutyrate and cellulose acetate phthalate blend microspheres	Providing a sustained drug release	Chaturvedi et al. (2013)
	*In vivo*	Albino rats	polyhydroxybutyrate and cellulose acetate phthalate blend microspheres	Increasing 5-FU anti-cancer effects	Chaturvedi et al. (2013)
Head and neck cancer	*In vitro*	Bovine mucosa	hydroxyethyl cellulose gel	Enhancing topical delivery of 5-FU to the buccal mucosa	Gratieri et al. (2014)

### 5.1 Colorectal cancer

In CRC point of view, there are a limited number of studies using nano-carriers based on cellulose. Yusefi and colleagues performed one of these studies and created a bionanocomposites through mounting Fe3O4 nanoparticles onto cellulose derived from rice straw (Yusefi et al., 2021b). Their analysis show that this carrier not only increases the anti-cancer effects of 5-FU but is also able to selectively deliver this drug to cancer cells rather than normal cells (Yusefi et al., 2021b). Another study provided microspheres which contained cellulose acetate phthalate (CAP) and tried to assess its efficacy *in vivo* and *in vitro* (Chaturvedi et al., 2013). They loaded 5-FU into a microsphere composed of CAP and polyhydroxybutyrate (PHB) and produced Spherical particles with the size of 44 ± 11 μm. They’re *in vitro* analysis showed a sustained release of 5-FU in HT-29 colon cancer cell (Chaturvedi et al., 2013). Furthermore, using this carrier on albino male Wistar rats in which were CRC was chemically induced. They detected lower levels of plasma albumin, creatinine, and a decrease in white blood cells as well as platelets (thrombocytopenia) were noted in animals treated with 5-FU microspheres compared to those given the standard 5-FU formulation. These results show that this carrier is effective for enhancing anti-tumor activities of 5-FU (Chaturvedi et al., 2013).

### 5.2 Breast cancer

Regarding breast cancer treatment, the most recent study is conducted by Jaisankar et al. (2024) in 2024 which developed polymeric nanofiber mats from some agents including methylcellulose, iron oxide (Fe_3_O_4_) nanoparticles, and polyethylene glycol. Their results show that along with high 5-FU encapsulation, these Nanofiber mats enhance anticancer effectiveness (78%) against MDA-MB 231 cancer cells. This indicates that a minimal quantity of the 5-FU drug (15.86%), combined with elevated levels of O_2_••, H_2_O_2_, and OH• radicals produced from Fe_3_O_4_, has catalyzed the Fenton reaction to eliminate the cancer cells within a brief period of 24 h (Jaisankar et al., 2024). This study was in continuation of their previous work in 2022 which assessed the drug release pattern of these nanofiber mats and showed that in a period of 16 days, 5-FU is released from this carrier in the cancer site (Jaisankar et al., 2022). In one of the most recent studies, Ghasemizadeh and colleagues utilized the water-in-oil-in-water (W/O/W) technique for the first time for producing a pH-responsive nanocomposite. Their carrier was based on halloysite nanotube (HNT) which was coated with carboxymethyl cellulose (CMC)/PEG hydrogel (Ghasemizadeh et al., 2023). They used this nano-carrier on MCF-7 breast cancer cells and detected that drug entrapment and loading can be highly enhanced through the usage of this carrier. Drug entrapment and loading had the rates of 46% and 87% respectively in this study in this study (Ghasemizadeh et al., 2023). The *in vitro* drug release tests also indicated that 5-FU was delivered more effectively and steadily in an acidic environment compared to a physiological setting, supporting the pH sensitivity of this newly developed nano-carrier (Ghasemizadeh et al., 2023). Although this nano-carrier seems promising, but some *in vivo* studies are required for confirming the efficacy of this method *in vivo*. Shamsabadipour and colleagues used W/O/W method for providing a nanocomposite composed of polyvinylpyrrolidone (PVP)/carboxymethyl cellulose (CMC)/γ-alumina (Shamsabadipour et al., 2023). Their analysis demonstrated the effective incorporation of 5-FU into the nanocarrier and verified the chemical bonding and crystalline characteristics of the synthesized nanocomposite (Shamsabadipour et al., 2023). Drug release analysis of their nan-carrier showed a pattern in which the drug was released in a controlled manner, maintaining over 96 h of retention. Additionally, the loading and entrapment efficiencies of this Nanoemulsion carriers were found to be 44% and 86%, respectively. Moreover, improved cytotoxicity and increased late-stage apoptosis were observed with the PVP/CMC/γ-alumina/5-FU combination. Over 90% cell viability was detected in this study, confirming the biocompatibility and biosafety of their nanocarrier. Furthermore, the porous structure of the PVP/CMC/γ-alumina allows this nanocarrier to utilize a high specific surface area, making it more responsive to changes in environmental conditions, such as pH (Shamsabadipour et al., 2023). Another study tried to create a hydrogel based on cellulose. They used cellulose nanofibers (CNFs) in combination with alginate/pectin and used this hydrogel on breast cancer cells (Balahura et al., 2021). They observed that when tumor cells were exposed to their carrier, a significant cytotoxic effect was observed, along with a rise in caspase-1 levels released into the culture media and increased production of reactive oxygen species (ROS). The amount of ROS produced was directly related to the concentration of the anti-tumor agent contained in the scaffolds (Balahura et al., 2021).

### 5.3 Other cancers

There is also a very limited number of investigations on the efficacy of cellulose-based nano-carriers. For instance, a study tried to provide a method for targeted topical delivery of 5-FU to head and neck cancers (Gratieri et al., 2014). They used iontophoresis of 5-FU, using an aqueous solution and a 2% hydroxyethyl cellulose gel at a pH of 7.6. They observed that iontophoresis led to a notable increase in the mucosal deposition of this drug when compared to passive diffusion (Gratieri et al., 2014). Furthermore, after 20 min of iontophoresis, there was approximately an 8-fold increase in 5-FU (Gratieri et al., 2014). They concluded that iontophoresis could facilitate the precise topical delivery of chemotherapy drugs to the buccal mucosa, potentially allowing for a less invasive treatment option for head and neck cancers (Gratieri et al., 2014). There are no other studies in this field which means these results showed be confirmed with further research.

Hemicellulose-derived carriers demonstrated the ability to enhance 5-FU encapsulation, provide sustained and pH-responsive release, and improve drug delivery selectivity to tumor cells. Reported anticancer actions included increased cellular uptake, prolonged retention at the tumor site, and induction of apoptosis, often through reactive oxygen species generation when combined with other functional materials. For example, cellulose nanofiber–alginate/pectin hydrogels significantly increased caspase-1 activity and ROS production in breast cancer cells, while Fe_3_O_4_-decorated cellulose matrices selectively enhanced cytotoxicity toward colorectal cancer cells in both 2D and 3D models. *In vivo*, cellulose acetate phthalate/polyhydroxybutyrate microspheres reduced biochemical toxicity markers such as elevated liver enzymes and myelosuppression compared with free 5-FU, while maintaining strong antitumor effects. Most *in vitro* studies reported minimal harm to normal cells or confirmed good biocompatibility (>90% viability) of the carriers. Overall, hemicellulose-based systems appear to potentiate 5-FU’s anticancer activity while mitigating systemic and off-target toxicities.

Overall, the reported effects of hemicellulose-based nanocarriers also vary across different cancer cell models, although the number of available studies remains more limited compared with chitosan systems. Most investigations have focused on colorectal cancer cell lines such as HT-29 and HCT-116, reflecting the relevance of polysaccharide-based materials for colon-targeted drug delivery. In these models, hemicellulose-based carriers have demonstrated improved cytotoxicity and more sustained intracellular exposure to 5-FU compared with the free drug, primarily due to controlled drug release and partial enzymatic degradation of the polysaccharide matrix. Studies involving other cancer types, including breast cancer cell lines such as MCF-7, are less common but still indicate enhanced antiproliferative effects when 5-FU is delivered through modified hemicellulose nanoparticles. However, because hemicellulose polymers are typically neutral or only weakly charged, their cellular uptake is generally less dependent on electrostatic membrane interactions and more influenced by nanoparticle size, surface functionalization, and receptor-mediated endocytosis. As a result, the magnitude of the cytotoxic response can vary more noticeably between different cancer cell types. Consequently, although hemicellulose-based delivery systems show promising potential for improving the therapeutic performance of 5-FU, the relatively small number of studies and limited comparative analyses across multiple cancer cell lines mean that the statistical reliability of these observations remains less robust, highlighting the need for broader biological evaluation in diverse cancer models.

### 5.4 Challenges and opportunities in hemicellulose-based 5-FU delivery systems

Although hemicellulose has emerged as a promising biopolymer for drug delivery due to its abundance, biocompatibility, and structural flexibility, several limitations currently hinder its widespread application in 5-FU-based cancer therapy. A key challenge is the structural heterogeneity of hemicellulose, which varies significantly depending on its botanical source and extraction method. This inconsistency impacts the reproducibility of nanoparticle synthesis and can lead to variability in drug encapsulation efficiency, degradation rate, and release kinetics. Furthermore, most existing studies are limited to *in vitro* evaluations, often using monolayer cancer cell models that do not accurately mimic the complexity of the tumor microenvironment. Consequently, the lack of robust *in vivo* studies limits the predictive value of preclinical findings and slows the path toward clinical translation (Barhoum et al., 2020; Ciolacu and Darie, 2016; Sandhya, 2023). Another limitation is the relatively poor mechanical stability of native hemicellulose nanoparticles, which may compromise their integrity under physiological conditions, particularly during systemic circulation. Crosslinking and chemical modification can enhance stability, but these approaches often introduce synthetic reagents or increase cytotoxicity, undermining the biocompatibility advantages of hemicellulose. Additionally, the absence of extensive toxicological profiling, especially regarding chronic administration or accumulation in off-target tissues, represents a significant barrier to regulatory approval (Barhoum et al., 2020; Ciolacu and Darie, 2016; Sandhya, 2023).

Nonetheless, hemicellulose offers notable opportunities for developing advanced 5-FU delivery platforms. Its abundant functional groups allow for easy chemical modification, enabling the creation of stimuli-responsive systems that release 5-FU in response to pH, enzymatic activity, or oxidative stress. Moreover, hemicellulose can be blended with other polymers, such as chitosan or polyethylene glycol, to improve mechanical strength and modulate release behavior. The potential for green synthesis and sustainable sourcing further enhances its appeal as a next-generation carrier. Moving forward, integrating hemicellulose into hybrid nanocomposites and validating its performance in complex biological models will be critical for unlocking its translational potential in oncology (Barhoum et al., 2020; Ciolacu and Darie, 2016; Sandhya, 2023).

## 6 5-Fluorouracil and lignin-based delivery systems

### 6.1 Lignin and different cancer types

Lignin is a biopolymer with high molecular weight (typically ranging from 1,000 to over 10,000 Da) which can be found in the cell walls of plants and has emerged as a promising material in drug delivery systems (Kumar et al., 2021). Its rich phenolic content allows for the modification and functionalization of lignin, enabling the development of innovative drug carriers. Lignin can be utilized to create nanoparticles or hydrogels that encapsulate therapeutic agents, enhancing their stability and controlled release. Lignin is not a single compound but rather a heterogeneous polymer composed of various phenolic compounds (Upton and Kasko, 2016). Its structure is primarily formed from three main aromatic alcohols, known as monolignols: P-coumaryl alcohol (H-unit), Coniferyl alcohol (G-unit), and Sinapyl alcohol (S-unit). These monolignols are linked together via a series of ether and carbon-carbon bonds, resulting in a three-dimensional network. The relative proportions of these units can vary significantly among different plant species and even within different tissues of the same plant, leading to variations in lignin structure and properties. The polymerization of lignin involves various types of linkages like β-O-4 Ether Linkages, α-O-4 Ether Linkages, and 5-5′ Linkages. The complexity arises from the random nature of these linkages, resulting in a highly branched and irregular structure that can be difficult to characterize fully. Similar to chitosan and cellulose, Lignin can also be used in drug delivery systems (Zhang et al., 2023).

**FIGURE 6:**
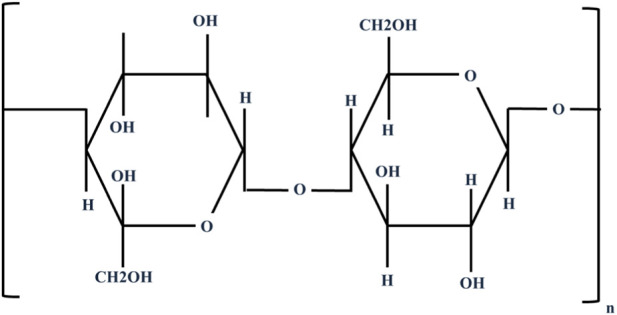
Hemicellulose structure. Cellulose consists of long, unbranched chains of β-D-glucose. The β(1→4) linkage means that each glucose unit is rotated 180° relative to its neighbors, allowing for a straight-chain conformation. The hydroxyl (-OH) groups on the glucose monomers participate in extensive hydrogen bonding both within and between cellulose chains. This hydrogen bonding contributes significantly to the stability and rigidity of the cellulose structure.

Jeyaraj et al. (2016) are one of the research groups which worked on this polymer. They separated lignin from Aloe barbadensis Miller and utilized it for the creation of a drug carrier through grafting with methacrylate (MA). The resulting lignin-grafted methacrylate (LIG-g-MA) was synthesized using atom transfer radical polymerization (ATRP). 5-FU served as a model anti-cancer medication and was encapsulated within the hollow nanocarrier using the emulsion/solvent evaporation method (Jeyaraj et al., 2016). They assessed the efficiency of the carrier by examining encapsulation efficiency and *in vitro* drug release. Additionally, the effect of 5-FU loaded onto this carrier on cell viability and cytotoxicity was tested on MCF-7 and VERO cell lines. They concluded that their carrier is effective for enhancing 5-FU encapsulation and increasing its cytotoxic effects against breats cancer cells (Jeyaraj et al., 2016) (Figure 6).

Lignin-based nanocarriers, though less extensively studied than chitosan or hemicellulose systems, have shown promising potential in enhancing 5-FU delivery. Their rich phenolic and functional group content allows for chemical modification, enabling sustained and targeted release. In the reviewed work, lignin-grafted methacrylate nanoparticles successfully encapsulated 5-FU and increased cytotoxicity against MCF-7 breast cancer cells, with greater selectivity compared to normal VERO cells. While comprehensive *in vivo* safety assessments are lacking, available *in vitro* data indicate favorable biocompatibility, with reduced toxicity toward non-malignant cells and potential intrinsic antioxidant effects that may further limit off-target damage. These findings suggest lignin-based carriers can amplify the therapeutic effects of 5-FU while offering a comparatively low toxicity profile, though further toxicological and pharmacokinetic evaluations are needed for clinical translation.

### 6.2 Challenges and opportunities in lignin-based 5-FU delivery systems

The application of lignin as a nanocarrier for 5-FU delivery represents a novel and environmentally sustainable direction in cancer nanomedicine. However, its practical implementation faces considerable scientific and technical hurdles. One of the primary challenges is the intrinsic chemical complexity and heterogeneity of lignin. As a highly branched, irregular polymer composed of various phenolic subunits, its structure is not only species-dependent but also significantly influenced by the extraction method, be it kraft, organosolv, or enzymatic hydrolysis. This inconsistency complicates the reproducibility of nanoparticle synthesis, affects drug-carrier interactions, and limits scalability (Pathania et al., 2023; Sipponen et al., 2019; Figueiredo et al., 2017). Moreover, lignin’s hydrophobic nature and limited aqueous solubility can restrict its utility in biological systems without prior chemical modification. Although various functionalization strategies, such as grafting with hydrophilic polymers or introduction of carboxyl and amine groups, have been explored to improve solubility and drug-loading capacity, these approaches often lack uniformity and require optimization on a case-by-case basis. In addition, limited data exist on lignin’s pharmacokinetics, biodegradability *in vivo*, and long-term biosafety, making it difficult to anticipate regulatory acceptance or clinical performance (Pathania et al., 2023; Sipponen et al., 2019; Figueiredo et al., 2017). Nevertheless, lignin’s unique biochemical properties open exciting avenues for innovation. Its rich content of aromatic and hydroxyl groups allows for multiple conjugation strategies, facilitating the development of pH-sensitive, redox-responsive, or enzyme-degradable delivery systems. Lignin also exhibits inherent antioxidant and antimicrobial properties, which may provide additional therapeutic benefits or reduce inflammation at tumor sites. Furthermore, lignin nanoparticles can serve as dual-purpose platforms, offering both therapeutic and diagnostic (theranostic) capabilities when combined with imaging agents (Pathania et al., 2023; Sipponen et al., 2019; Figueiredo et al., 2017). As research progresses, integrating lignin with more established biopolymers or inorganic nanomaterials may help overcome its individual limitations while preserving its advantages. Future studies focusing on standardization, *in vivo* degradation, and immune interactions will be essential to fully realize lignin’s promise in targeted 5-FU delivery for cancer therapy (Pathania et al., 2023; Sipponen et al., 2019).

## 7 Comparative analysis of biopolymer-based nanocarriers for 5-FU delivery

**FIGURE 7:**
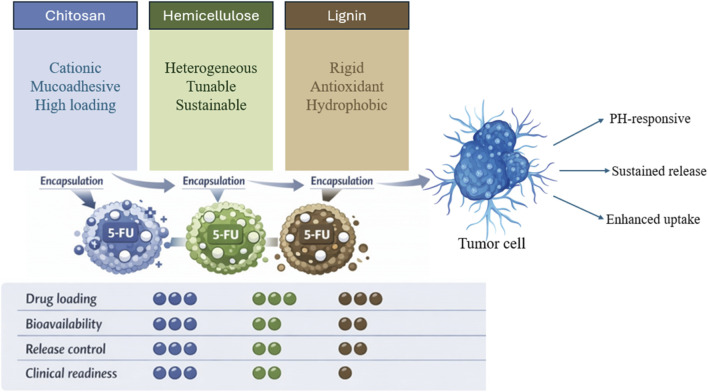
Comparative overview of chitosan-, hemicellulose-, and lignin-based nanocarriers for 5-fluorouracil (5-FU) delivery. The schematic illustrates the structural characteristics and functional differences among three major biopolymer platforms used in 5-FU nanocarrier design. Chitosan (cationic polysaccharide), hemicellulose (heterogeneous plant-derived polysaccharide), and lignin (aromatic biopolymer) are depicted with their key physicochemical properties influencing drug delivery performance. Each biopolymer is shown forming 5-FU-loaded nanoparticles through encapsulation processes, followed by delivery to tumor cells. The primary mechanisms of action include pH-responsive release, sustained drug release, and enhanced cellular uptake. A comparative summary highlights relative trends in drug loading capacity, bioavailability, release control, and clinical readiness across the three platforms. Overall, these nanocarriers contribute to improved therapeutic outcomes by enhancing efficacy, prolonging drug action, reducing systemic toxicity, and potentially lowering dosing frequency.

A key objective of this review is to provide a system-level comparison of chitosan-, hemicellulose- and lignin-based nanocarriers for the delivery of 5-FU. While all three platforms demonstrate the capacity to enhance the pharmacological performance of 5-FU, meaningful differences exist in their physicochemical characteristics, drug loading behavior, release kinetics, bioavailability, therapeutic efficacy, and translational potential. It should be noted, however, that direct quantitative comparisons across studies are limited by variations in experimental design, model systems, and formulation strategies. Therefore, the following analysis emphasizes consistent trends and relative performance rather than absolute values (Figure 7).

From a structural and physicochemical standpoint, chitosan-based systems exhibit clear advantages due to their cationic nature, which promotes strong interactions with negatively charged cell membranes and mucosal surfaces. This property enhances cellular uptake and contributes to improved drug internalization. Hemicellulose-based carriers, while biodegradable and chemically versatile, display significant variability in composition depending on their botanical source, which can influence reproducibility and mechanical stability. Lignin-based systems offer structural rigidity and intrinsic antioxidant properties due to their aromatic backbone; however, their hydrophobicity and limited aqueous solubility necessitate chemical modification for effective biomedical application.

In terms of drug loading and encapsulation efficiency, chitosan-based nanocarriers consistently demonstrate high loading capacities, often attributed to electrostatic interactions and ease of chemical functionalization. Hemicellulose-based systems generally achieve moderate to high encapsulation efficiencies, particularly when formulated as composite hydrogels or nanofibrous matrices, though variability remains a concern. Lignin-based carriers exhibit moderate drug loading capacity, with fewer studies available and less consistency across formulations, reflecting their earlier stage of development. Differences become more pronounced when evaluating drug release kinetics and duration of therapeutic action. Chitosan-based systems frequently exhibit pH-responsive and sustained release profiles, enabling preferential drug release within the acidic tumor microenvironment. This controlled release behavior often extends drug exposure over several hours to days, reducing the need for frequent dosing. Hemicellulose-based carriers similarly provide sustained and, in some cases, prolonged release profiles, with certain formulations maintaining drug release over extended periods (e.g., multiple days). However, their release kinetics are often more dependent on chemical modification or composite design. Lignin-based systems primarily demonstrate sustained release behavior; however, fine control over release kinetics remains less developed, limiting their ability to precisely match tumor-specific conditions.

Bioavailability is a critical parameter where chitosan-based systems show the most consistent improvement. Their mucoadhesive properties and ability to transiently open tight junctions enhance drug absorption and retention, leading to increased local and systemic bioavailability depending on the route of administration. Hemicellulose-based carriers can improve bioavailability through sustained release and protection of the drug from premature degradation, although their lack of intrinsic mucoadhesive properties may limit absorption efficiency unless modified. Lignin-based systems may contribute to improved stability of 5-FU and reduced premature degradation; however, direct evidence of enhanced bioavailability remains limited and requires further investigation.

With respect to therapeutic efficacy, all three platforms demonstrate improved anticancer activity compared to free 5-FU, primarily through enhanced cellular uptake, sustained drug exposure, and targeted delivery. Chitosan-based nanocarriers provide the most robust and consistent evidence of enhanced cytotoxicity against cancer cells, along with reduced toxicity to normal tissues, supported by both *in vitro* and *in vivo* studies. Hemicellulose-based systems also show promising anticancer effects, particularly in hybrid or composite formulations, though the supporting evidence is more limited and largely restricted to *in vitro* models. Lignin-based carriers have demonstrated encouraging cytotoxic effects in early studies, but their efficacy data remain sparse and require validation in more complex biological systems. An important clinical implication of these differences is their potential impact on dosage requirements and treatment duration. By improving drug stability, enhancing tumor targeting, and enabling sustained release, chitosan-based nanocarriers may allow for reduced dosing frequency and lower total drug doses while maintaining therapeutic efficacy. Hemicellulose-based systems may offer similar advantages in extending drug exposure and reducing dosing intervals, although this is highly dependent on formulation design. In contrast, while lignin-based systems show potential for sustained delivery, insufficient data currently exist to determine their impact on dosing strategies or long-term treatment regimens.

When comparing these three platforms from a clinical translation perspective, it becomes clear that their readiness is determined not only by biological compatibility but also by technological scalability, regulatory maturity, and translational evidence supporting human use. Chitosan-based formulations occupy the leading position among natural polymer carriers for 5-FU because several chitosan derivatives and nanoparticulate systems have reached advanced preclinical phases, and some related chitosan formulations are already used as excipients in approved pharmaceuticals. Extensive toxicological and biodistribution data show low immunogenicity, rapid clearance of degradation fragments via renal filtration, and minimal long-term accumulation, which collectively de-risk early clinical trials. Chitosan offers precise control over particle size (typically 100–400 nm) and surface charge through its degree of deacetylation, enabling reproducible production under Good Manufacturing Practice (GMP) standards. For 5-FU delivery, it allows pH-triggered release and tumor-targeted accumulation through the enhanced permeability and retention (EPR) effect, and can also be conjugated with folate or transferrin ligands to achieve active targeting of overexpressed receptors in colorectal and breast cancers. In a translational context, chitosan carriers are suited for oral and injectable formulations, offering options to reduce systemic toxicity by localizing 5-FU action. However, their solubility limitations near neutral pH and variability in polymer grade remain obstacles to regulatory approval as an active nanocarrier system rather than a simple excipient. To move into Phase I human studies, standardized chitosan batches with validated physicochemical specifications and reproducible nanoparticle formation kinetics will be essential, parameters that the FDA and EMA increasingly require to deem natural-polymer nanomedicines “chemically defined” (Divya and Jisha, 2018). In terms of *in vivo* validation, chitosan nanocarriers have undergone numerous animal studies, especially in murine xenograft models of colorectal, breast, and gastric cancers. These animal studies frequently report statistically significant improvements in tumor suppression, reductions in systemic toxicity, and controlled pharmacokinetic behavior compared with free 5-FU. The statistical robustness of this evidence varies: many studies use modest sample sizes (n = 5–10 per group), which limits power, but the consistency of results across many independent laboratories strengthens the reliability of the overall conclusions. Importantly, while several chitosan derivatives and chitosan-containing formulations have been evaluated clinically as excipients or wound-healing materials, no human clinical trials have yet tested chitosan-based 5-FU nanocarriers. Overall, the accumulated literature on chitosan is statistically more reliable than that for hemicellulose or lignin, primarily because of (a) the large number of independent studies, (b) frequent reproduction of findings across species and disease models, and (c) the presence of structured toxicological assessments. Despite the absence of human trials for 5-FU systems, the extensive animal data offer a strong translational foundation. Variability in chitosan source and degree of deacetylation remains a limitation, but the quantity and reproducibility of existing data make chitosan the most clinically mature platform among the three.

Hemicellulose-based nanocarriers sit at an intermediate stage between concept and translation. Their abundant hydroxyl and acetyl groups enable versatile chemical derivatization, permitting the engineering of redox-responsive, enzyme-degradable, or thermosensitive release systems for 5-FU. While the sustainability and eco-friendly manufacturing benefits of hemicellulose are compelling, clinical translation remains limited largely by insufficient structural standardization and mechanical robustness of the resulting nanoparticles. Hemicellulose extracted from different botanical sources exhibits variable branch composition (arabinoxylan vs. glucomannan), which affects polymer chain interactions, hydrophilicity, and drug-loading capacity, all parameters that must be tightly controlled in a clinical-grade material. Hybridization with chitosan or polyethylene glycol has proven effective for improving particle stability, release kinetics, and *in vivo* dispersion, producing composite nanocarriers that could deliver 5-FU with predictable pharmacokinetics. For clinical advancement, hemicellulose systems must undergo formal toxicological and immunological evaluation because plant polysaccharides can elicit mild innate immune responses via toll-like receptor activation. In addition, hemicellulose’s biodegradation by gut microflora suggests a promising route for targeted colorectal cancer therapy using oral delivery of 5-FU, potentially reducing systemic exposure, an idea that warrants pharmacokinetic and pharmacodynamic studies in animal models prior to Investigational New Drug (IND) submission. Therefore, hemicellulose may not be “clinic-ready” today but could progress rapidly if efforts focus on polymer purification, molecular weight standardization, and reproducible nanocomposite synthesis to meet GMP quality benchmarks. Most studies focus on chemical modification, hybrid nanocomposite formation, and stimuli-responsive behavior rather than full therapeutic application. Within this set, only a small subset (likely fewer than ten detailed reports) specifically examines hemicellulose systems for 5-FU delivery. This limited number reflects the platform’s early developmental stage but also highlights its potential for innovation, particularly in creating biodegradable, environmentally sustainable carriers.

Regarding biological evaluation, hemicellulose nanocarriers have undergone very few animal studies, and none involve 5-FU-loaded systems. Existing *in vivo* work typically focuses on safety or biodistribution rather than anticancer efficacy and often uses small sample sizes, reducing statistical power. As a result, the current evidence base lacks the depth necessary to draw reliable statistical conclusions about therapeutic benefit, pharmacokinetics, or toxicity. Importantly, no human studies have evaluated hemicellulose nanocarriers for any anticancer application, and none are registered for clinical trial consideration in 5-FU delivery. Given the low number of animal studies and the absence of human data, the statistical reliability of hemicellulose research remains moderate to low, not because results are negative, but because the sample size of the literature is small. The variability of hemicellulose composition between botanical sources further complicates standardization, limiting cross-study comparability. Nonetheless, early findings consistently show good biocompatibility and favorable degradation characteristics, suggesting that once systematic animal-level testing begins, the platform could advance significantly in clinical credibility. Lignin-based platforms currently represent the most experimental yet potentially transformative alternative thanks to their distinctive physicochemical and biological features. Lignin’s rigid aromatic backbone provides exceptional stability against enzymatic degradation and oxidative stress, while its diverse phenolic and aliphatic functional groups permit extensive chemical grafting for controlled drug release or responsive behavior under tumor microenvironment conditions. The study by Jeyaraj et al. demonstrated how atom transfer radical polymerization could graft methacrylate moieties onto lignin isolated from Aloe barbadensis, creating a lignin-g-methacrylate nanocarrier that encapsulated 5-FU efficiently and enhanced cytotoxicity against MCF-7 breast cancer cells without comparable toxicity toward VERO normal cells—compelling preliminary proof of concept. Complementary reports describe green-synthesized lignin nanoparticles for targeted delivery to cancer cells and lignin-based microcarriers showing enhanced antiproliferation *in vitro*. Despite these promising observations, lignin systems face substantial translational challenges: heterogeneity arising from different plant sources and extraction processes complicates standardization, and inconsistent molecular mass distributions hinder precise control of particle formation and drug-release profiles. Furthermore, the lack of comprehensive toxicology, biodistribution, and long-term metabolism studies prevents regulatory agencies from evaluating lignin nanomaterials confidently for human use. Nevertheless, lignin introduces valuable opportunities for engineering multifunctional, stimuli-responsive carriers, for example, redox-sensitive delivery exploiting the ROS imbalance in tumors or co-delivery of 5-FU with antioxidant phytochemicals to reduce off-target toxicity.

Clinically, successful translation of lignin systems would likely begin with topical or localized applications (e.g., intratumoral hydrogel composites) where systemic exposure is limited, before progressing to systemic routes following full toxicological validation. Future development should emphasize standardized lignin fractions, integration with approved polymers (such as PEG or chitosan), and comprehensive cytotoxicity assays under GMP synthesis conditions to satisfy regulatory expectations for natural-polymer nanomedicines. Jeyaraj et al. (2016) reports specific evaluation of lignin-g-methacrylate as a 5-FU carrier, and only a limited number of additional publications exist examining lignin nanoparticles for anticancer drugs more broadly. Despite the relatively small literature base, these studies highlight lignin’s unique chemical versatility, antioxidant properties, and ability to undergo functionalization, positioning it as a promising next-generation nanocarrier system. In terms of biological testing, lignin nanocarriers have undergone very few animal studies, most of which focus on safety, biodistribution, or antioxidant activity rather than anticancer efficacy. The 5-FU-based lignin system described in your manuscript has been validated only *in vitro*, with enhanced cytotoxicity against MCF-7 breast cancer cells but no *in vivo* confirmation. Sample sizes in the existing animal studies are typically small and heterogeneous in design, limiting the statistical reliability and generalizability of current findings. Critically, no human studies have evaluated lignin nanocarriers of any type for cancer therapy. Consequently, the statistical reliability of lignin research is low at the clinical-translation level, primarily because the number of studies is small and most lack comprehensive toxicological or pharmacokinetic evaluation. Structural heterogeneity of lignin from different biomass sources introduces additional variability that makes cross-study comparisons difficult. Still, the consistency of favorable *in vitro* results, including the enhanced 5-FU encapsulation and biological activity reported in Jeyaraj et al., suggests strong scientific potential. To advance toward clinical relevance, lignin systems will require standardized extraction chemistry, larger-scale animal trials, and systematic toxicology to establish a safety profile acceptable to regulators.

Overall, chitosan is closest to real clinical translation because of established safety records, scalable processing, and well-defined behavior *in vivo*, placing it as the logical first candidate for 5-FU nanocarriers to enter human trials (Table 3). Hemicellulose presents a sustainable, adaptable next-generation system whose unique biodegradation profile aligns well with localized gastrointestinal cancer therapy, but its progression depends on achieving reproducible molecular characterization and validating *in vivo* efficacy. Lignin, while scientifically remarkable for its multifunctional chemistry and intrinsic bioactivity, remains the furthest from clinical readiness and requires systematic toxicological testing, structural uniformity, and pilot pharmacokinetic studies before any Phase I evaluation can occur.

**TABLE 3:** Comparison between chitosan-based, hemicellulose-based, and lignin-based systems.

Parameter/Feature	Chitosan-based platform	Hemicellulose-based platform	Lignin-based platform
Chemical nature	Linear cationic polysaccharide derived from chitin; forms protonated amino groups enabling electrostatic interactions	Branched neutral or weakly acidic heteropolysaccharide composed of xylose, mannose, and arabinose units; chemically tunable through derivatization	Cross-linked aromatic biopolymer rich in phenolic and methoxy groups; amphiphilic behavior due to mixed hydrophobic/hydrophilic segments
Surface charge and hydrophilicity	Positively charged → strong electrostatic attraction to negatively charged cell membranes	Usually neutral or slightly negative; charge modifiable via acetylation or carboxylation	Neutral to slightly negative; aromatic surface allows π–π or hydrophobic interactions with biomolecules
Particle size range (nm)	Typically, 50–200 nm (controllable by ionic gelation and pH)	80–250 nm depending on polymer crosslinking and substituents	100–300 nm; heterogeneity arises from variable lignin source and polymerization method
Drug-loading capacity (5-FU)	Moderate–high (∼25–60%) via ionic interactions and hydrogen bonding	Moderate (∼20–40%) through hydrogen bonding and physical entrapment	High potential (∼30–70%) through aromatic stacking and graft copolymer interaction
Release behavior	pH-responsive; faster release in acidic tumor microenvironment due to protonation	Controlled and tunable by chemical modification; enzymatically degradable	Generally sustained release governed by hydrophobic domains and cross-link density
Biodegradation	Readily enzymatically degraded; biocompatible and non-toxic	Gradual enzymatic degradation by hemicellulases; good biodegradability	Slower degradation; variable depending on lignin modification; minimal human data
Interaction with cancer cells	Enhanced cellular internalization through charge-mediated uptake; strong cytotoxicity in HT-29, HeLa, MCF-7	Good compatibility and retention in colon-derived cancer cells (e.g., HCT-116); moderate uptake in others	Potent oxidative and apoptotic response in breast cancer cells (MCF-7); limited data for other lines
Therapeutic strengths	Proven biocompatibility, reproducibility, established preclinical record; scalable production	Structural tunability, environmentally sustainable source, suitable for targeted oral delivery	Unique antioxidant and functional properties, high payload capacity, potential synergistic activity
Major limitations	Poor solubility at neutral pH; batch-to-batch variability due to degree of deacetylation	Limited mechanical robustness; insufficient toxicological and *in vivo* evidence	Source heterogeneity; lack of standardization and safety/toxicology data
Preclinical/clinical maturity	Advanced preclinical; numerous animal studies; no human trials for 5-FU nanoparticles	Early preclinical; very few *in vivo* studies; no clinical trials	Emerging experimental stage; mostly *in vitro* evidence only; no *in vivo* or clinical studies

In strategic terms, a tiered translational pathway can be envisioned (Miura et al., 2010): chitosan-5-FU systems proceed toward near-term human trials (Longley et al., 2003), hemicellulose hybrids advance through animal pharmacology and formulation optimization, and (Farokhzad and Langer, 2009) lignin nanocarriers are refined through chemical standardization and biosafety profiling to prepare for long-term clinical innovation. Integration of these biopolymers into a unified platform could ultimately yield multicomponent nanomedicines capable of combining chitosan’s permeability, hemicellulose’s tunability, and lignin’s chemical reactivity—an evolution fully consistent with ongoing trends in green and personalized oncology.

The biological performance of polymeric nanocarriers depends strongly on their physicochemical characteristics, which influence cellular uptake, intracellular trafficking, and drug-release behavior. Consequently, chitosan-, hemicellulose-, and lignin-based nanocarriers interact differently with various cancer cell lines, resulting in distinct cytotoxic responses. Chitosan-based nanocarriers generally demonstrate enhanced cellular uptake in multiple cancer cell models, including breast (MCF-7), cervical (HeLa), and colorectal (HT-29) cell lines. This behavior is largely attributed to the cationic nature of chitosan, which promotes electrostatic interactions with negatively charged phospholipids and glycoproteins on cancer cell membranes. These interactions facilitate adsorptive endocytosis and improve intracellular delivery of 5-FU. As a result, chitosan nanoparticles frequently produce stronger cytotoxic responses compared with free drug formulations in several epithelial cancer models. However, the magnitude of this effect varies among cell lines depending on membrane composition, receptor expression, and metabolic sensitivity to 5-FU. For example, colorectal cancer cells often exhibit pronounced susceptibility due to their inherent sensitivity to antimetabolite drugs such as 5-FU. Hemicellulose-based nanocarriers display more variable interactions across cancer cell types, primarily because hemicellulose polymers are typically neutral or weakly charged unless chemically modified. Their cellular uptake therefore relies less on electrostatic attraction and more on particle size, surface functionalization, and receptor-mediated endocytosis pathways. In several reported systems, hemicellulose carriers have demonstrated promising activity in colon-derived cancer cell lines, where enzymatic degradation of polysaccharides within the tumor microenvironment may facilitate drug release. However, compared with chitosan systems, hemicellulose nanoparticles often exhibit moderate internalization efficiency, which may lead to more gradual cytotoxic responses in certain cell models. Lignin-based nanocarriers exhibit a distinct interaction mechanism related to their aromatic and phenolic structure. The hydrophobic domains and phenolic groups present in lignin nanoparticles can promote interactions with cellular membranes and intracellular proteins, potentially contributing to oxidative stress–related cytotoxic effects. In the lignin-grafted methacrylate system reported by Jeyaraj et al., enhanced cytotoxicity was observed in MCF-7 breast cancer cells following delivery of encapsulated 5-FU. This effect was attributed to improved drug encapsulation and sustained intracellular release. Nevertheless, the available evidence remains limited to a small number of *in vitro* studies, and comparative data across multiple cancer cell lines are still scarce. Taken together, these observations highlight clear differences in how the three biopolymer platforms interact with cancer cells. Chitosan nanocarriers benefit from strong electrostatic membrane interactions that enhance uptake in many epithelial cancer cell lines. Hemicellulose systems rely more heavily on structural modification and enzymatic degradation to achieve effective drug release, resulting in more variable cellular responses. Lignin nanocarriers represent an emerging platform whose aromatic structure may enable unique intracellular interactions but whose biological effects remain less thoroughly characterized. These distinctions emphasize the importance of selecting carrier materials according to the biological characteristics of the target cancer type.

## 8 Clinical translation and regulatory considerations of biopolymer-based nanocarriers

Although considerable advancements have been made in designing biopolymer-based nanocarriers for cancer treatment, only a limited number of these systems have successfully progressed toward clinical use. Among naturally derived polymers, chitosan has demonstrated the most substantial advancement in biomedical applications, largely due to its recognized biocompatibility, biodegradability, and well-documented safety profile. It has been extensively investigated as a delivery vehicle for chemotherapeutic drugs, genetic materials, and protein-based therapeutics, with several formulations reaching advanced stages of preclinical development. Conversely, nanocarriers derived from hemicellulose and lignin remain primarily in early experimental phases. Nonetheless, emerging research underscores their structural adaptability, adjustable physicochemical characteristics, and inherent biological properties, including antioxidant and immunomodulatory activities. Translating these nanocarrier systems from laboratory research into clinical settings presents several major challenges. A central issue is the inherent variability associated with natural polymers. Factors such as molecular weight distribution, degree of deacetylation (for chitosan), branching complexity in hemicellulose, and compositional heterogeneity in lignin can differ depending on the source material and extraction procedures. This variability complicates large-scale manufacturing, consistent quality assurance, and regulatory compliance—each of which is essential for pharmaceutical development. Consequently, establishing standardized purification, chemical modification, and analytical characterization protocols is critical to ensuring reproducibility and facilitating clinical advancement.

Safety and pharmacokinetic considerations also require careful evaluation. While many natural polymers are generally considered biocompatible, systemic administration of nanoparticulate systems necessitates thorough assessment of biodistribution, metabolic pathways, and potential long-term toxicity. Characteristics such as particle size, surface charge, and chemical functionalization can markedly influence circulation duration, tissue accumulation, and immune system interactions. Therefore, comprehensive toxicological and immunological investigations are essential, particularly for cancer therapies that involve repeated dosing schedules. Regulatory approval pathways add another layer of complexity. Nanomedicine products may be classified differently such as pharmaceuticals, biologics, or combination products, depending on their composition and therapeutic mechanism. This classification variability can extend development timelines and requires extensive documentation of critical quality attributes, including particle size uniformity, drug encapsulation efficiency, release behavior, and storage stability. Accordingly, the development of standardized analytical techniques and scalable manufacturing platforms is fundamental for regulatory acceptance.

Despite these obstacles, several approaches may facilitate clinical progress. Rational design and polymer engineering can enhance consistency and performance by precisely controlling structural features, such as chitosan’s degree of deacetylation or the functional group density of lignin-based materials. Surface modification strategies—incorporating targeting moieties, polyethylene glycol (PEG), or stimuli-responsive linkers—can improve tumor-specific accumulation, extend systemic circulation, and enable controlled drug release within the tumor microenvironment. Furthermore, advancements in scalable production methods, including microfluidic technologies and continuous nanoprecipitation systems, offer promising routes for industrial-scale fabrication while preserving particle uniformity. Future developments are likely to benefit from integrating biopolymer nanocarriers with established therapeutic modalities. For instance, these systems can be engineered for the simultaneous delivery of chemotherapeutics and immunomodulatory agents, potentially amplifying anticancer immune responses. Combining nanocarrier-based drug delivery with immunotherapy, radiotherapy, or molecularly targeted treatments may further enhance therapeutic efficacy. In parallel, the increasing focus on personalized medicine suggests that biomarker-driven patient stratification and tumor-responsive delivery platforms could optimize treatment outcomes. In summary, although considerable translational barriers remain, chitosan, hemicellulose, and lignin possess distinct advantages—including renewability, biodegradability, and versatile chemical functionality—that position them as promising materials for next-generation drug delivery technologies. Ongoing efforts aimed at material standardization, rigorous safety assessment, and scalable production will be pivotal in advancing these innovative systems from experimental research to clinical reality.

## 9 Limitations

Despite the promising therapeutic performance of chitosan-, hemicellulose-, and lignin-based nanocarriers for 5-FU delivery, several critical limitations hinder their clinical translation. Scalability remains a major challenge. Fabrication techniques such as ionic gelation, nanoprecipitation, and chemical crosslinking are typically optimized at laboratory scale and are difficult to reproduce under industrial manufacturing conditions. Parameters including mixing dynamics, solvent removal, and crosslinking efficiency can significantly influence particle size, polydispersity, and drug loading. These issues are particularly pronounced for lignin and hemicellulose due to their structural complexity and variability, complicating process standardization and large-scale reproducibility. Reproducibility is further limited by the intrinsic heterogeneity of natural polymers. Variations in molecular weight, degree of deacetylation (in chitosan), branching patterns (in hemicellulose), and monolignol composition (in lignin) can lead to substantial differences in physicochemical properties and biological performance. This variability directly affects drug encapsulation efficiency, release kinetics, and cellular interactions, making cross-study comparisons and regulatory validation challenging. Regulatory barriers also represent a significant constraint. While chitosan has a relatively well-established safety profile and is used in certain biomedical applications, hemicellulose- and lignin-based systems lack comprehensive toxicological, pharmacokinetic, and long-term safety data. Regulatory approval requires detailed characterization of degradation products, immunogenicity, and biodistribution, as well as adherence to GMP standards. The absence of standardized characterization protocols for these nanocarriers further complicates regulatory evaluation.

Another key limitation is the insufficient translation from *in vitro* to *in vivo* systems. Although many studies report enhanced cytotoxicity and controlled drug release *in vitro*, these results do not consistently predict *in vivo* behavior. Factors such as protein corona formation, immune recognition, off-target accumulation, and rapid clearance can significantly alter therapeutic outcomes. This gap is particularly evident for lignin-based nanocarriers, where *in vivo* data remain scarce. Finally, formulation stability and shelf-life present additional challenges. Nanocarriers may undergo aggregation, premature drug leakage, or chemical degradation during storage, which can compromise efficacy and safety. Ensuring long-term stability while maintaining functional performance is essential for clinical and commercial viability. Addressing these limitations requires the development of standardized extraction and characterization methods, scalable and reproducible manufacturing processes, and comprehensive *in vivo* and pharmacokinetic studies. Early integration of regulatory considerations will be critical to facilitate the successful translation of these biopolymer-based nanocarriers into clinical applications.

## 10 Conclusion and future aspects

Lignin, chitosan, and cellulose are three environmentally safe and compatible biopolymers that boasts several beneficial features, such as significant biocompatible and antioxidant capabilities. These organic polymers can be modified using various chemical techniques to create a variety of nanomaterials that hold potential for use in biomedicine. These natural polymers offer excellent biocompatibility, biodegradability, and functional versatility, making them ideal candidates for next-generation drug delivery systems. This review highlights recent advancements in nanocarriers which are provided based on these polymers for cancer treatment through delivering 5-FU. Studies show that these nanocarriers are able to increase the efficacy of 5-FU on many cancer cells including breast, colorectal, and head and neck cancers (Mazandarani et al., 2023; Jaisankar et al., 2022; Jeyaraj et al., 2016). This effect is possible through increasing drug entrapment and encapsulation and providing a sustained drug release in the cancer site which enhances the cytotoxic effects of 5-FU. Advanced drug delivery systems (DDS) built on these polymers utilize various mechanisms, such as pH-sensitivity, enzymatic responsiveness, and active targeting via surface modifications (e.g., aptamer conjugation), to achieve controlled drug release and precise tumor targeting. Chitosan-based hydrogels and nanoparticles, for instance, have shown the ability to prolong drug retention at the tumor site while reducing exposure to healthy tissues. Hemicellulose and lignin carriers also demonstrate sustained drug release and compatibility with other polymers, enhancing encapsulation efficiency and mechanical stability. These systems outperform conventional drug delivery routes (e.g., intravenous, oral) by improving pharmacokinetics and reducing dose-limiting side effects.

Furthermore, the mucoadhesive and cationic nature of chitosan facilitates cellular uptake and permeability across biological membranes. Hemicellulose’s natural reactivity and tunable structure enable it to form hybrid composites for synergistic delivery. Lignin, despite its structural complexity, offers antioxidant properties and abundant functional groups for modification, allowing for redox-responsive or enzyme-degradable drug release profiles. Nevertheless, challenges such as scalability, batch-to-batch consistency, and incomplete pharmacokinetic profiling must be addressed before these nanocarriers can be translated into clinical practice. Standardization of synthesis protocols and comprehensive *in vivo* studies, including biodistribution, immunogenicity, and toxicity evaluations, are crucial next steps. Among the natural polymer-based nanocarriers reviewed, chitosan systems currently stand out for their higher encapsulation efficiencies, adjustable particle size, surface functionalization flexibility, and well-documented mucoadhesive properties, all of which contribute to improved tumor targeting and reduced systemic exposure of 5-FU. Hemicellulose-based carriers offer a renewable and chemically versatile platform, particularly for hybrid systems, though improvements in structural stability and large-scale processability are still needed. Lignin-based nanocarriers present unique antioxidant activity and a high density of functional groups for stimuli-responsive drug release, but remain less explored in preclinical models compared to chitosan and hemicellulose. Overall, chitosan appears to be the most developed toward translational readiness, while hemicellulose and lignin remain promising but require further optimization and validation before clinical consideration. Evidence across the reviewed studies indicates that chitosan-, hemicellulose-, and lignin-based carriers can substantially improve the therapeutic index of 5-FU by enhancing tumor-selective delivery, prolonging drug release, and reducing systemic toxicity. Chitosan systems demonstrated consistent anticancer activity through mechanisms such as pH-responsive release, improved cellular uptake, and apoptosis induction, while sparing normal cells and lowering biochemical toxicity markers *in vivo*. Hemicellulose-derived carriers achieved high encapsulation efficiencies, sustained or stimuli-responsive release, and selective tumor targeting, with *in vivo* studies showing reduced hematologic and hepatic toxicity compared to free 5-FU. Lignin-based platforms, though less studied, also enhanced 5-FU cytotoxicity toward cancer cells while maintaining good biocompatibility, with early evidence of selectivity against malignant over normal cells. Collectively, these findings support that biopolymer-based nanocarriers not only enhance the anticancer potency of 5-FU but also mitigate its dose-limiting toxicities, underscoring their promise for safer, more effective cancer chemotherapy.

However, the lack of human studies in this field is limiting the application of these nano-carriers in clinical practice and therefore, more investigations on chitosan, cellulose, and lignin are required. Furthermore, 5-FU is used in treating many gastrointestinal cancers including gastric, esophageal, and pancreatic cancers which are not investigated. We suggest that using these nano-carriers on these cancers might be promising for decreasing the mortality and morbidity of these cancers. In conclusion, the strategic application of chitosan-, hemicellulose-, and lignin-based nanocarriers holds substantial promise for revolutionizing 5-FU delivery in oncology. These systems not only improve drug performance but also align with sustainable and green chemistry approaches. Continued interdisciplinary research, including robust clinical validation, is essential to unlock their full therapeutic potential and bring them closer to clinical use in managing colorectal, breast, and other solid tumors.
